# New Drug Repositioning Candidates for T-ALL Identified *Via* Human/Murine Gene Signature Comparison

**DOI:** 10.3389/fonc.2020.557643

**Published:** 2020-11-09

**Authors:** Raphaël Bonnet, Marielle Nebout, Carine Brousse, Frédéric Reinier, Véronique Imbert, Pierre Simon Rohrlich, Jean-François Peyron

**Affiliations:** ^1^ Université Côte d’Azur, INSERM, C3M, Nice, France; ^2^ Pediatric Hematology-Oncology, CHU de Nice, Nice, France

**Keywords:** T-cell acute lymphoblastic leukemia, PTEN tumor suppressor gene, conserved genes, CMAP, drug-repositioning

## Abstract

T-cell Acute Lymphoblastic Leukemia (T-ALL) is an aggressive subtype of leukemia for which important progress in treatment efficiency have been made in the past decades to reach a cure rate of 75%–80% nowadays. It is nevertheless mandatory to find new targets and active molecules for innovative therapeutic strategies as relapse is associated with a very dismal outcome. We designed an experimental workflow to highlight the conserved core pathways associated with leukemogenesis by confronting the gene expression profiles (GEPs) of human T-ALL cases to the GEP of a murine T-ALL representative model, generated by the conditional deletion of the *PTEN* tumor suppressor gene in T cell precursors (tPTEN-/-). We identified 844 differentially expressed genes, common GEPs (cGEP) that were conserved between human T-ALL and murine signatures, and also similarly differentially expressed, compared to normal T cells. Using bioinformatic tools we highlighted in cGEPan upregulation of E2F, MYC and mTORC1. Next, using Connectivity Map (CMAP) and CMAPViz a visualization procedure for CMAP data that we developed, we selected *in silico* three FDA-approved, bioactive molecule candidates: α-estradiol (α-E), nordihydroguaiaretic acid (NDGA) and prochlorperazine dimaleate (PCZ). At a biological level, we showed that the three drugs triggered an apoptotic cell death in a panel of T-ALL cell lines, activated a DNA damage response and interfered with constitutive mTORC1 activation and c-MYC expression. This analysis shows that the investigation of conserved leukemogenesis pathways could be a strategy to reveal new avenues for pharmacological intervention.

## Introduction

Treatments for acute lymphoblastic leukemia (ALL) are improving as the overall survival rate at five years from diagnosis raised from 12% in 1960 to 65% in 2014 and around 80% nowadays (1). Unfortunately, in the case of T-cell Acute Lymphoblastic Leukemia (T-ALL), an aggressive subtype of leukemia which represents 15% of ALL cases, this success is more mitigated compared to B-ALL. Very few genetic prognostic markers can be used for the risk stratification of T-ALL, preventing any adaptation of treatment strategies (2). Therapeutic protocols for T-ALL are very complex and highly toxic. In most cases, in the induction phase of the treatment, patients are given a 4-drug cocktail associating a glucocorticoid (dexamethasone) with an anthracycline (daunorubicin) to interfere with DNA replication, a poison of the mitotic spindle (vincristine) to interfere with cell division and an anti-metabolite enzyme (L-asparaginase) (3, 4). Dramatically, relapse can still occur in 15 to 20% of pediatric T-ALL patients who then face a dismal outcome with at best only a 6-month survival for half of them as no effective therapeutic options exist at this stage. A better deciphering of the mechanisms of relapse in T-ALL is therefore mandatory to discover new therapeutic strategies (5, 6). T-ALL display multiple genetic lesions that define different molecular subgroups with different risk and aggressiveness (7–9). Nevertheless T-ALL cases share several common dis-regulations in signaling pathways regulating proliferation, survival and interactions with their supportive and protective microenvironment. For instance, a constitutive activation of the PI3K-AKT-mTORC1 axis is observed at a high frequency (85%) to stimulate leukemic growth (10–12). An aberrant activation of the ligand-activated transcription factor NOTCH1, that is crucial for T cell development is observed in 60-75% of T-ALL (7–9). Also, 70% of T-ALL have lost the *P16/INK4A*, P19/ARF tumor suppressors leading to unrestrained stimulation of cyclin-CDK complexes and cell cycle progression on one arm and inactivation of the p53 tumor suppressor response on the other. These dysregulated events are being investigated as targets of future treatments (5, 13). In parallel, *ex vivo* drug profiling to evaluate the chemosensitivity of relapse samples could be another powerful approach to propose new therapeutic options for some T-ALL subgroups or individual patients (14).

Cancer remains a most complex biological system with an important plasticity allowing an escape from the effects of chemotherapeutic drugs to produce relapse. Nevertheless, most processes that define cell states such as cell division, apoptosis or senescence have been conserved in evolved organisms and this could be the same for cancer. We thought to determine the cancer-associated processes that are conserved across human T-ALL and a murine T-ALL model to untangle cancer complexity at its core. We thus compared the gene expression profiles of human T-ALL samples present in public databases to that of a in-house murine T-ALL model generated by the thymocyte-specific deletion of the *PTEN* tumor suppressor (15). It allowed us to highlight the conserved differentially regulated genes and to determine through bioinformatic enrichment tools the cellular functions that are associated with leukemia and conserved between the two models. Then, we took advantage of the Connectivity Map (CMAP) computational pipeline from The Broad Institute (https://www.broadinstitute.org) to identify *in silico* potential active molecules. We selected three compounds: α-estradiol (α-E), nordihydroguaiaretic acid (NDGA), and prochlorperazine dimaleate (PCZ) whose potential anti-leukemic effect was then explored at a biological level.

## Material and Methods

### Data Processing and Differential Expression Analysis

Normalized gene expression datasets were retrieved from Gene Expression Omnibus (GEO: https://www.ncbi.nlm.nih.gov/gds/). Gene expression profiles (GEP) of 13 T-ALL patients samples and 17 control healthy samples (T cells) were studied from GSE48558. GEP from the tPTEN-/- mouse genetic T-ALL model was obtained from GSE39591 (16). A GEP Linear Models for Microarray Data (LIMMA) method was used to reveal differentially expressed genes (pValue<0.05) using the web interface Phantasus (17) (**Supplementary Table 1**). Gene lists were processed using R and biomaRt library to match mice gene symbols to human gene symbols after conversion and to identify the conserved differentially expressed genes (cDEG) between the two species. Symbols of conserved genes that vary in the same direction in the two models are available in **Supplementary Table 2**. The same method was used to analyze cDEG between dataset GSE117165 (PHF6 KO mouse) and GSE48558 (T-ALL), **Supplementary Tables 1** and **6**. Significativity of the DEG overlap was assessed through Fisher exact test implemented in GeneOverlap R package (18) based on DEG symbols from the two models and average genome size.

### Enrichment Analysis and *In Silico* Drug Screening

To extrapolate from genes to cellular mechanisms we used the R package fGSEA (19) to calculate the enrichment of the dysregulated genes in GSEA (Gene Set Enrichment Analysis) hallmarks and reactome pathways. For the fGSEA on reactome database, we selected the first 10 most enriched processes. Also we queried the Gene Ontology database through the R package GOplot (20). We applied a pValue and qValue cutoff at p**<**0.001 and queried all ontologies. All enrichment results are available in **Supplementary Table 3**.

For the *in silico* identification of bioactive compounds we used CMAP with the top 1000 HG-U133a probes for both up and down regulated genes in our signatures. We selected only the results with negative enrichment scores with at least 3 molecules in a batch on the HL60 Acute Myeloïd Leukemia (AML) cell line which was chosen as a representative of hematologic malignancies in CMAP. We reasoned that although T-ALL are of lymphoïd lineage and HL60 of myeloïd origin the two types of leukemia could have a close gene signature. The complete output table of molecules provided by CMAP is available in **Supplementary Table 5**.

### Bibliometric Score and Graphical Representation (CMAPViz)

To help the selection of molecules to be tested within the CMAP hit molecule list we implemented a bibliometric score based on three criteria, 1: the number of clinical trials (c) filed at https://clinicaltrials.gov/ and related to acute lymphoblastic leukemia, 2: the number of publications (p) referring to the hit molecule (m) in association with leukemia on PubMed and finally 3: a score ranging from 1 to 3 proportional to the best impact factors (i) for the previously mentioned association. Additionally a weight (w) of 2 was given to the final score to enhance the visualization of the score. Details of scores can be found in **Supplementary Table 4**.

$$Bibliometric\;score_{m}=\log(c_{m}*\;p_{m}*i_{m}*w)$$

In order to visualize the results in a more user-friendly manner we developed a tool called CMAPViz available as a R package. This representation allows the user to visualize more efficiently the best candidates resulting from the *in silico* drug screening.

### Cell Culture

The human T-ALL cell lines were grown in RPMI 1640 medium supplemented with 10% (JURKAT, MOLT-16) or 20% (PEER, LOUCY, ALL-SIL) Fetal Calf Serum (FCS) and 50 U/ml penicillin, 50μg/ml streptomycin, and 1mM sodium pyruvate. The murine primary KO99L cell line was established by cultivating tumoral thymocytes from a tPTEN-/- mouse, in RPMI 1640 supplemented with 10% FCS, 50 U/ml penicillin, 50μg/ml streptomycin, and 1mM sodium pyruvate. All cell lines were grown at 37°C under 5% CO2.

### Drugs and Reagents

RPMI 1640 media, penicillin, streptomycin, sodium pyruvate, and fetal calf serum (FCS) were purchased from Life Technologies (Courtaboeuf, France). Antibodies against c-MYC (#9402S), pSer235/236-S6RP (#4856), S6RP (#2217S) were from Cell Signaling (Danvers, MA, USA), HSP90 (sc-13119) from Santa Cruz Biotechnology (Santa Cruz, CA, USA). α-Estradiol (E8750) and NDGA (74540) were from Sigma-Aldrich, St. Louis, MO. PCZ was purchased from Tocris Bio-Techne (3287, Minneapolis, MN, USA).

### Analysis of Cell Viability

In a 96-well plate, 40,000 cells per well were incubated with effectors for 48 h at 37°C (n=3 per experiment). Cell death was assessed after staining with 1µg/ml of DAPI (4,6 diamidino-2-phenylindole) followed by FACS analyses on MACSQuant10^®^ analyzer (Miltenyi Biotec, Bergisch Gladbach, Germany). Living cells were quantified as the percentage of DAPI negative cells. All statistical data from functional assays are presented as the mean ± s.d.

### Annexin V/DAPI Staining Assay

Apoptosis assessment was performed by measuring phosphatidyl-serine membrane redistribution *via* annexin-V-FITC staining (130-093-060, Miltenyi Biotec). After incubation with effectors for indicated times, cells were re-suspended in staining solution and incubated in the dark (15 min, RT). Then, cells were re-suspended in DAPI staining solution (1 μg/mL) and immediately analyzed by FACS on a MACSQuant10^®^ analyzer (Miltenyi Biotec). For blocking apoptosis cells were preincubated with 20µM Q-VD-OPh (A1901, CliniSciences, Nanterre, France) for 1 h and were then washed with PBS 1X and treated with α-Estradiol, NDGA and PCZ at 8 µM, 42 µM, and 10 µM respectively +/- Q-VD-OPh.

### Western Blotting

After treatment, cells were solubilized in lysis buffer (50 mM HEPES, pH 7.4, 150 mM NaCl, 20 mM EDTA, 100 mM NaF, 10 mM Na3VO4, 1 mM PMSF, 1mM aprotinin and 0.5% NP40) and incubated for 30 min at 4°C. Lysates were centrifuged at 12,000 g for 15 min at 4°C and supernatants were adjusted with Laemmli SDS sample buffer. Proteins were separated by SDS/PAGE and transferred to ECL membranes (GE Healthcare, Velzy-Villacoublay, France) in a Tris (20 mM), glycine (150 mM), and ethanol (20%) buffer at 100 V for 1 h at 4°C. Antibodies were incubated in saturation buffer (50 mM Tris pH 7.5, 50 mM NaCl, 0.2% Tween, 5% BSA) and revealed with a secondary-peroxidase-conjugated antibody followed by ECL detection (GE Healthcare). Protein bands were quantified using the ImageJ software. The signal of a given total protein was normalized to the signal of Hsp60, used as a loading control from the same sample. Bands corresponding to phosphorylated proteins were normalized to the signal of the corresponding total protein which had been normalized to Hsp60.

### Statistics

GraphPad Prism 7 was used for data analysis and to determine IC_50_ for each drug. Results are presented as the mean ± SD for at least three repeated independent experiments.

## Results

### Identification and Enrichment Analysis of Conserved Genes in Murine and Human T-ALL

**Figure 1:**
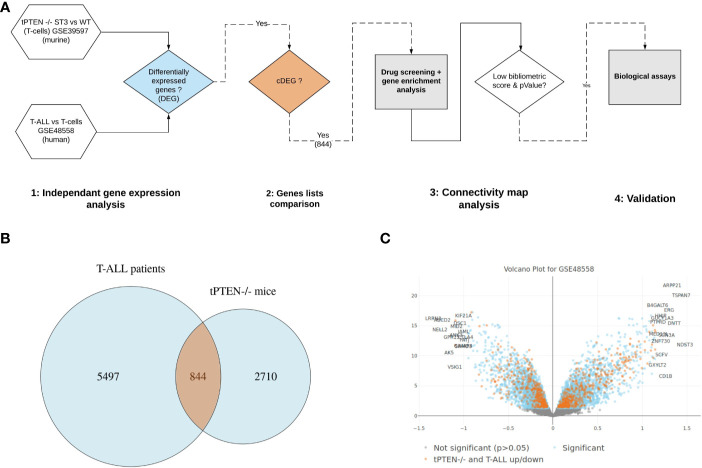
Identification of common differentially expressed genes in human and murine T-cell Acute Lymphoblastic Leukemia (T-ALL). **(A)** Bioinformatic analysis pipeline. **(B)** Differentially expressed genes overlapping between human and mice datasets. **(C)** volcano plot of human T-ALL dataset (GSE48558). Differentially expressed genes highlighted in orange represent the common core genes, that are similarly regulated (up/down) between mouse and human datasets (cDEGs).

Our whole gene analysis pipeline is reported on **Figure 1A**. We confronted transcriptomic data obtained from human T-ALL gene signatures present in public datasets to the one we established from a tPTEN-/- T-ALL mouse model, to highlight the gene dysregulations that could be shared between the two models. We first extracted the differentially expressed genes (DEG) between leukemic cells and their normal counterparts, both for the murine T-ALL model (GSE38597) and the human T-ALL samples (GSE48558), to identify 3,554 and 6,341 DEG (p<0.05) from mouse and human datasets respectively. The similarity of the two DEG from the two species was found statistically significant (overlap: 1,355 genes, Fisher exact test p=2e-31). Then, by comparing the murine and human lists, we identified among the 1,355 overlapping DEG, 844 common differentially expressed genes (cDEG) (**Figure 1B**) that were similarly differentially expressed (in the same direction: up/up, down/down, between the human and murine DEGs, **Figure 1C**). Using data from tPTEN-/- cells could be a bias as *PTEN* mutations are observed in only 15%–20% of the cases, although it has to be kept in mind that the PI3K/Akt/mTOR pathway negatively controlled by PTEN is abnormally active in almost 90% of T-ALL cases. We used a data set (GSE117165) corresponding to a transgenic mouse model expressing an oncogenic form of NOTCH1, an event observed in more than 60% of T-ALL. We extracted 491 cDEG with the human T-ALL dataset (compared to 844 when using tPTEN-/- cells) available in **Supplementary Table 6**. The two lists were compared by an hypergeometric test to give an excellent p value of 3.8e-12 demonstrating that the overlap of 59 genes between the two cDEGs is significant. We could therefore extrapolate that using transcriptomic data from the tPTEN-/- model could be relevant for a global study of T-ALL leukemogenesis.

### Bioinformatic Analysis of T-ALL cDEG

We next used several bioinformatics tools to visualize the biological processes and functions behind these 844 genes.

**Figure 2:**
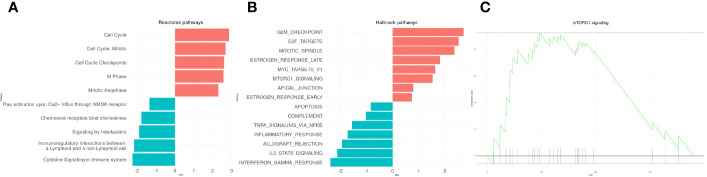
Visualization of enriched reactome and hallmarks genes sets in T-cell Acute Lymphoblastic Leukemia (T-ALL)-cDEG. The 10 top enriched reactome **(A)** and hallmark **(B)** gene sets are presented. **(C)** Enrichment plot of the mTORC1 signaling pathway.

We first run a GSEA analysis on reactome pathways (**Figure 2A**) and hallmarks processes (**Figure 2B**) to reach a better resolution on the biological processes associated with leukemogenesis. The analysis showed the up-regulation of cell cycle, mitosis, M phase, anaphase. This was evidenced at a molecular level with the positive enrichment of G2M checkpoint genes, targets of E2F and MYC, that are well known to be associated with cell transformation (**Figure 2B**). Also, the mTORC1 signaling pathway frequently upregulated in many cancers including acute leukemias was found positively enriched (NES=2.87, p=0.006) (**Figures 2B, C**). The results also provided evidence for the down-regulation of immune functions and cytokines signaling. Genes relevant for TNFα/NF-κB signaling, IL2, STAT5 signaling, inflammatory responses, IFNγ responses were also negatively modulated. All 127 up-regulated processes and 47 down-regulated processes are reported in **Supplementary Table 3**.

**Figure 3:**
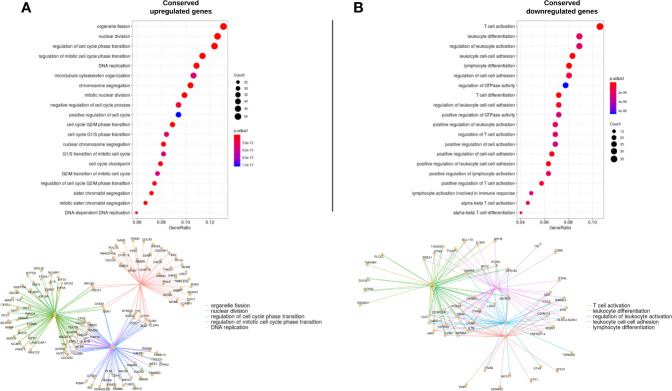
Gene ontology enrichment analysis representing the enriched processes for up-regulated (**A**, left column) and down regulated (**B**; right column) genes. The top 20 enriched processes are displayed. The size of each point represents the number of genes that enrich in the corresponding process. The x-axis value represents that number in the form of a gene-ratio. The lower part of each column represents the genes enriched in the top 5 processes and their functional links.

Then, a GO enrichment analysis (p<0.001) was run with the cDEGs that were either up- or down-regulated. Not surprisingly, up-regulated genes enriched in processes appeared related to cell proliferation such as cycle transition phases and cell division (G2/M phases transition, chromosome segregation, DNA replication) (**Figure 3A**). On the opposite, down-regulated genes demonstrated a decrease in T-cell activation, differentiation and adhesion (**Figure 3B**). All 144 up-regulated processes and 68 down-regulated processes and their corresponding genes are reported in **Supplementary Table 3**.

### Drug Query Using CMAP and Identification of Drug Repositioning Candidates Through CMAPViz

**Figure 4:**
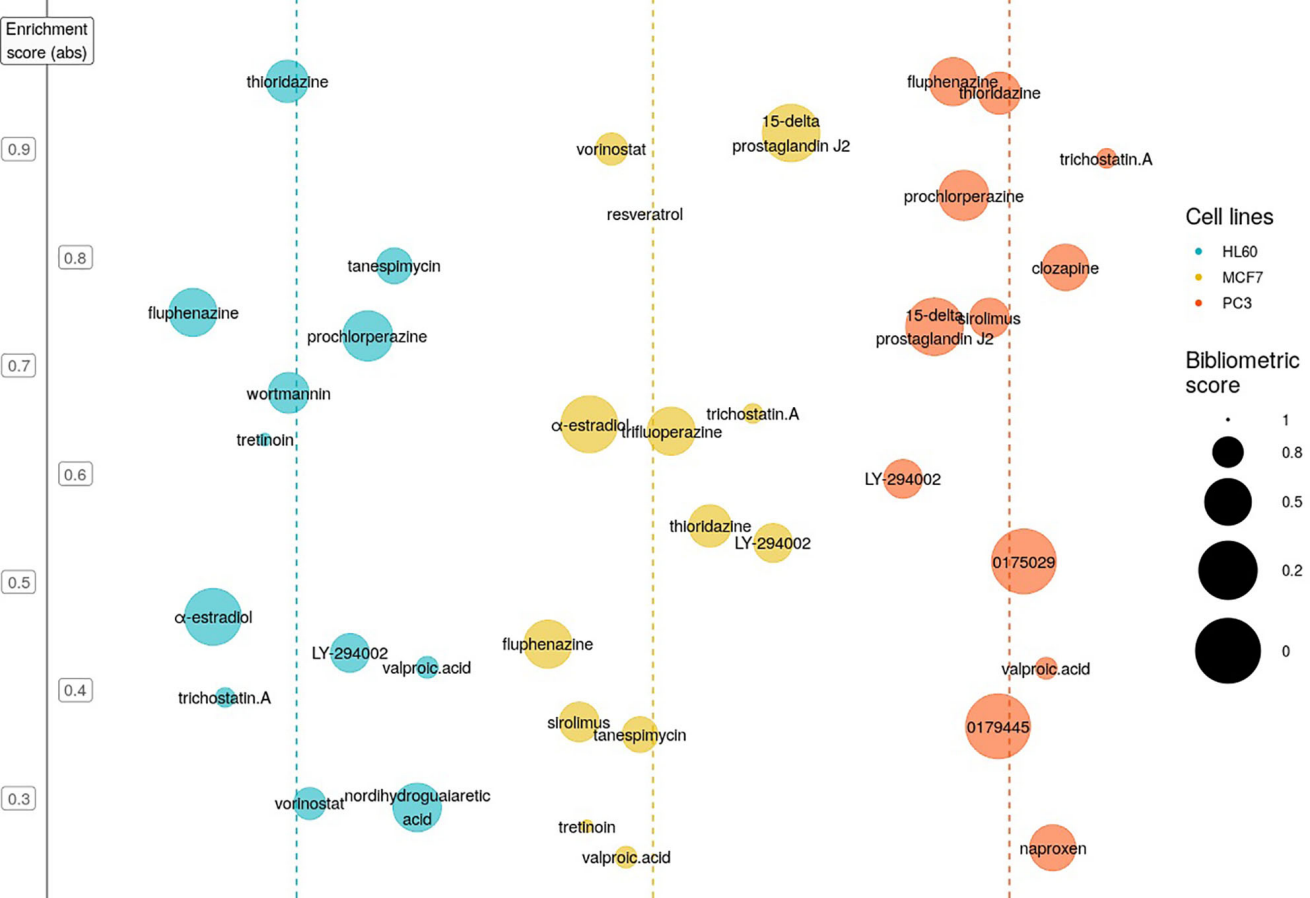
Selection of potentially T-cell Acute Lymphoblastic Leukemia (T-ALL) active molecules using Connectivity Map (CMAP) and CMAPViz. CMAPViz bubble plot for the top hit molecules resulting from CMAP analysis after applying statistical cut offs. Hit molecules are sorted horizontally by cell line data (blue: HL60, yellow: MCF7 and orange: PC3) and vertically by absolute enrichment value. We applied horizontal jittering to avoid overlap in the representation. The size of the circle for each molecule is inversely proportional to the bibliometric score.

We applied the CMAP gene pattern matching algorithm (21) to our T-ALL cDEG signature in order to search *in silico* for molecules that could target the conserved mechanisms of T-ALL leukemogenesis. CMAP is a database of gene expression signatures produced by molecules/drugs/perturbagens acting on a panel of cell lines that are representative of human cancers such as MCF7 for breast cancer, PC3 for prostate cancer, HL60 for leukemia. We searched in CMAP for molecules with a negative enrichment score (ES), meaning that they generated gene signatures on HL60 that negatively correlated with our T-ALL cDEG query signature. By reversing gene expression associated with leukemogenesis, we made the assumption that these molecules would trigger T-ALL cell death (**Figure 4**). Then, because most of the molecules listed in CMAP database have been thoroughly studied, even in T-ALL, we decided to implement a bibliographic score that takes into account, and for each molecule: i) the number of publications referring to the hit molecule, ii) its relationship to acute lymphoblastic leukemia, iii) the number of leukemia-related clinical trials it is involved in, and iv): the impact factor of the publications. Finally, we selected 3 molecules of interest: α-Estradiol (α-E) (n= 3, ES= −0.477), NDGA (n= 3, ES= –0.291), PCZ (n= 4, ES= −0.554) that present the lowest bibliometric score, underlying that they were scarcely described on leukemia. Thanks to the CMAPViz visualization tool we developed, it was possible to easily identify candidate drugs that immediately stood out from other drugs. While α-Estradiol had a negative ES, estradiol (17ß-estradiol, E2) showed a positive ES: 0.576 (HL-60, n=8), suggesting that the ß estrogen isomer has an opposite response to α-E by mimicking the cDEG response. To further ensure that our results are not driven by PTEN deletion but reflects more globally the core processes at play in T-ALL, we queried the CMAP database using the cDEG derived from a NOTCH1 oncogenic mouse model (GSE117165) compared to T-ALL (GSE48558). We obtained similar results (i.e: negative enrichment score) for α-E and PCZ. The results are shown in **Supplementary Table 7**.

### α-Estradiol, NDGA, and Prochlorperazine Induce Cell Death in T-ALL Cell Lines

**Figure 5:**
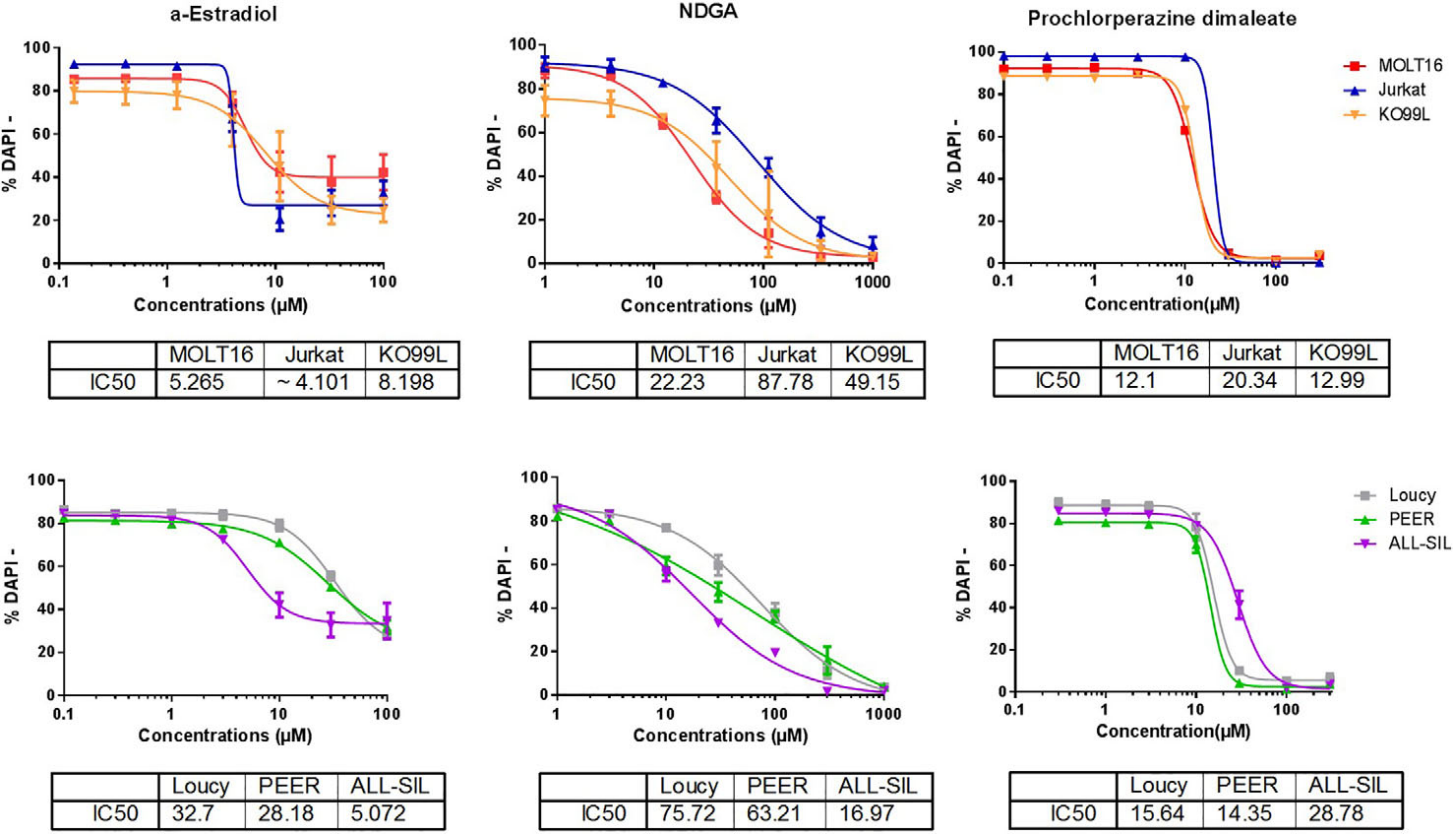
α-Estradiol, Nordihydroguaiaretic acid, and Prochlorperazine induce cell death in T-cell Acute Lymphoblastic Leukemia (T-ALL) cell lines. The effects of the three molecules were analyzed after a 48 h treatment of five human T-ALL cell lines: MOLT-16, Jurkat, LOUCY, PEER, ALL-SIL, and one murine line: KO99L. T-ALL cell viability was measured by flow cytometry after DAPI staining. The IC_50_ for each drug on the different cell lines are displayed in µM below each panel. The Figure represents the merge of three independent experiments. Results are presented as mean ± SD.

We first examined the anti-leukemic effects of α-E, NDGA, and PCZ in five different human T-ALL cell lines: MOLT-16, Jurkat, Loucy, PEER, ALL-SIL and on a murine T-ALL cell line (KO99L) that originated from a tPTEN-/- KO mice. After 48 h of incubation, the three molecules were capable to induce a dose dependent cell death in the six cell lines tested with different sensitivities, as visualized by flow cytometry after DAPI staining (**Figure 5**). α-E appeared slightly the most efficient molecule (IC_50_ ranging from 4.1 to 32.7µM; mean=13.9µM) before PCZ (12.1–28.7; 17.3 µM), then NDGA (16.9–87.8; 45.8) µM.

As controls, we tested on the MOLT-16, Jurkat and ALL-SIL lines, the action of one positive and one negative control molecules, respectively 17-AGG and Netilmicin. Tanespimycin/17-AAG has a negative enrichment score (ES= −0.799, n=12) on our T-ALL cDEG signature. 17-AAG had already been shown to have anti-leukemic properties (22). We observed that 17-AAG induced a dose dependent cell death in the three cell lines (**Supplementary Figure 1**). Netilmicin was selected because of its positive ES (1, n=1) corresponding to drugs that rather mimics the query signature in CMAP. We found that netilmicin was totally neutral on the three T-ALL cell lines (**Supplementary Figure 1**). We also observed in the CMAP database that α-E down-regulated all the cDEG genes that were found positively enriched in early and late estrogen response datasets (**Figure 2B**), except FASN (**Supplementary Figure 2** and **Supplementary Table 8**).

### α-Estradiol, NDGA, and Prochlorperazine Induce Apoptotic Cell Death

**Figure 6:**
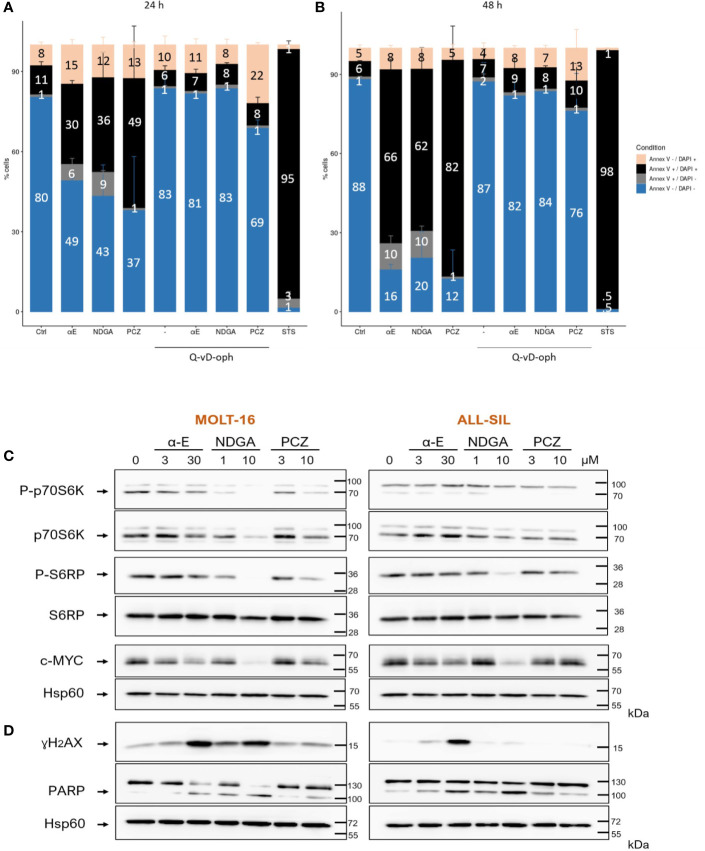
α-Estradiol, Nordihydroguaiaretic acid, and Prochlorperazine induce cell death and affect cellular signaling in T-cell Acute Lymphoblastic Leukemia (T-ALL) cell lines.**** **(A, B)** Apoptosis was measured by flow cytometry following annexin V-FITC/DAPI staining after 24 h **(A)** and 48 h **(B)** of incubation with or without 20 µM of the pan-caspase inhibitor Q-VD-OPh. **(C)** Western blot analysis of the effects of α-E, nordihydroguaiaretic (NDGA) and prochlorperazine dimaleate (PCZ) on mTORC1 activation and cMyc protein levels, 18 h after incubation with drugs. mTORC1 activation was measured through the analysis of p70S6K and S6RP phosphorylation. **(D)** DNA damage was assessed through the appearance of γH2AX, while apoptosis was evaluated through the cleavage of PARP. Arrows point to the specific bands of interest. Numbers on the right represent the molecular weight of SDS-PAGE markers in kilo Daltons (kDa). Protein band on Western blot were quantified using the ImageJ software. The signal of a given total protein was normalized to the signal of Hsp60, used as a loading control from the same sample. Bands corresponding to phosphorylated proteins were normalized to the signal of the corresponding total protein which had been normalized to Hsp60. The results are displayed on **Supplementary Figure 4**.

We next investigated on the MOLT-16 line which appeared globally, the most sensitive line to the three drugs, the possibility that the drugs could induce leukemic death through apoptosis. For this, cells were treated with drugs alone or after a 1 h preincubation with the pan-caspase inhibitor Q-VD-OPh. After 24 h (**Figure 6A**) or 48 h (**Figure 6B**) the cells were then stained with Annexin V-FITC/DAPI and analyzed by flow cytometry. A strong cell death response induced by the drugs was observed at 24 h (mean 38.3% annexin-V^+^-DAPI^+^ cells corresponding to a 3.4 fold induction compared to untreated cells). After 48 h, a mean of 70% of cells were found dead and the three drugs showed comparable death inducing capacity. Caspase inhibition almost completely prevented cell death induction by the three molecules thereby demonstrating an implication of caspases in the mode of action of the three drugs. The cytometry histograms of the effects of the selected drugs are displayed on **Supplementary Figure 3**. Staurosporine used as a positive control, triggered a massive death response (98%).

Apoptosis was further demonstrated by following through Western blotting the cleavage of PARP, a main substrate for the executioner caspase 3. The three drugs triggered the appearance of the 89kDa cleaved form of PARP, after 18 h (**Figure 6D**). NDGA was the more efficient (mean 4.2 fold induction) compared to α-E (3.1 fold) and PCZ (2 fold) (**Figure 6D**). Note that the measure of apoptosis was more sensitive through flow cytometry analysis of annexin-V binding (**Figures 6A, B**) than with PARP cleavage.

### The Effects of α-Estradiol, NDGA and PCZ Involve the DNA Damage Pathway

We analyzed the expression of γH2AX, a marker of DNA damage (**Figure 6D**). α-E and NDGA triggered the expression of γH2AX in the two cell lines with a respective mean of 13 and 2.3 fold induction. ALL-SIL cells appeared highly sensitive to α-E with a 21.5 fold γH2AX induction. By contrast, PCZ had little effect (mean 1.2 fold). The quantification of the protein bands is shown on **Supplementary Figure 4**.

In a cell cycle analysis we did not detect significant effects of any of the three drugs, used at concentrations below their death inducing dose (**Supplementary Figure 5**).

### α-Estradiol, NDGA, and PCZ Affect mTORC1 Signaling and c-MYC Levels in the MOLT-16 T-ALL Cell Line

T-ALL are characterized by a constitutive activation of the mTORC1 pathway which frequently occurs after inactivation of the *PTEN* tumor suppressor to sustain leukemic growth. Besides, leukemia also display at a high frequency an up-regulation of the c-MYC proto-oncogene that supports proliferation and survival. We analyzed the effects of the three drugs on these two important pathways. As shown on **Figure 6C**, the three drugs could dose dependently decrease the phosphorylation levels of both p70S6K and its substrate S6RP in both MOLT-16 and ALL-SIL cell lines. NDGA appeared the most active (mean 3 fold decrease for P-p70S6K and 1.75 fold for P-S6RP), followed by PCZ (P-p70S6K: 2.8 fold, P-S6RP: 1.75) then α-E (P-p70S6K: 1.7 fold, P-S6RP: 1.5 fold). MYC levels were also decreased by NDGA (5 fold) and α-E (1.8 fold) in the two cell lines, while PCZ only affected c-MYC (1.5-fold) in MOLT-16 cells. The quantification of the protein bands is displayed on **Supplemental Figure 4**. These results show that the selected drugs interfere with two important leukemogenesis pathways which likely participates in their anti-leukemic effects.

## Discussion

In this article, we compared datasets of gene expression profiles from human and murine leukemic samples with the aim to identify common pathways associated with leukemogenesis. There are many possible sources of heterogeneity in data production because of differences in sample handling and processing, in the sequencing technology used, and in this case in the species studied. Despite this, we showed that the differentially expressed genes (DEG) we identified in either human T-ALL patient samples and in a tPTEN-/- deficient T-ALL murine model (compared to their normal counterparts) were highly statistically similar, suggesting the existence of conserved core leukemogenesis mechanisms. Within 1,355 DEG that overlap on the two types of data, we found 844 common differentially expressed genes (cDEG) which had their expression similarly affected by leukemia development in both human and murine situations. We made the assumption that these cDEG could reflect the core leukemogenesis events at play and therefore could be used to find new targeting strategies for T-ALL.

Bioinformatic analyses highlighted the dysregulation of several important functions in leukemic cells. Indeed, T-ALL cells of both human and murine origin have an upregulation of genes participating in cell cycle progression *via* regulation of G1/S and G2/M transitions, M phase, chromosome segregation and DNA replication. At a molecular level, E2F targets, MYC targets, mTORC1 signaling were found upregulated. Our T-ALL cDEG analysis also reveals functions and genes which are downregulated in leukemic cells: cytokine signaling, inflammatory responses through TNF/NF-κB activation, IL2 and STAT5 signaling, interferon gamma response. Genes involved in apoptosis as well as genes participating in T cell differentiation and migration were also down-regulated in T-ALL. These observations fit with what is already known of T-ALL pathogenesis (8, 9), but the precise identification of the genes involved in these dysregulations could help reveal new directions for future T-ALL treatments (5).

We used this T-ALL cDEG to search for active molecules against T-ALL using the CMAP bioinformatic resource of the Broad Institute. We limited our *in silico* selection to the HL60 cell line that is representative of hematologic malignancies in CMAP and as an acute leukemia, the closest model in CMAP to T-ALL although from a different lineage (myeloïd vs lymphoïd). We selected drugs with a negative enrichment score (ES), meaning that they generated gene signatures that negatively correlated with our T-ALL cDEG query signature. By reversing gene expression associated with leukemogenesis, it is envisioned that these molecules could trigger T-ALL cell death. Moreover, to search from the outcome of CMAP for original compounds not yet described for leukemia, we implemented a bibliographic score to introduce a selection allowing us to limit the number of potential molecules to be tested. So the three molecules we selected: α-Estradiol (α-E), NDGA, and PCZ had rarely been associated with leukemia, in neither publications nor clinical trials. Their enrichment values were relatively low, because only few concentration values have been reported for them (α-E: n=3; NDGA: n= 3; PCZ: n=4) in the CMAP database on the HL60 cell line, opposite to more widely studied drugs such as the histone deacetylase inhibitors trichostatin A (n=34) and valproic acid (n=14). We believe that CMAP is a powerful hypothesis-generating tool and that biological evaluation of the selected drugs is crucial.

Our three anti-T-ALL candidate molecules have different modes of action. α-Estradiol (α-E) is a ligand for estrogen receptors, thoroughly studied in the case of breast cancer (BC). In particular BC cells expressing estrogen receptors (ER^+^BC) rely on them for proliferation, and as a consequence ER^+^ patients benefit from anti-estrogen treatment. 17ß-estradiol/E2 was shown to support glioblastoma cells proliferation and survival (23).

On the other side, estradiols have been shown to interfere with cancer cell proliferation in several cases. A recent high throughput screening (HTS) approach in T-ALL identified 2-methoxyestradiol (2-ME) as an active compound to target pre-leukemic stem cells (pre-LSCs) (24). 2-ME is a metabolite of 17ß-estradiol that displayed anti-proliferative properties in various cancers, although it’s mechanisms of action that may be independent of ER remain unclear, involving disruption of microtubules, blockade of angiogenesis and induction of apoptosis (25, 26). Interestingly, in this study (24), 2-ME was found to decrease the levels of c-MYC and of SCL, an effect that could potentially antagonize the dependency that pre-LSCs have on both SCL and the NOTCH-1 signaling pathway that activates c-MYC. 2-ME was also reported to inhibit proliferation and invasion of melanoma cells (27). Engagement of ERβ receptors on Hodgkin lymphoma cell lines by the DPN agonist inhibits their growth after autophagy induction (28). Another study showed that the anti-estrogen tamoxifen (TAM) had toxic effects on the human T-ALL cell line Jurkat and could partially bypass their resistance to dexamethasone used to treat T-ALL (29). Although counter-intuitive at first glance, this study could suggest that the anti-leukemic effects of estradiol and tamoxifen could be mediated through the G protein-coupled Estradiol Receptor (GPER) and not *via* classical ER, that are not bound by TAM and not expressed in several T-ALL cell lines (29). While TAM is an antagonist of ERαβand ERβ receptors it is an agonist of GPER. Along the same line, it was also reported that α-E through GPER1 could inhibit the spreading of granulosa cell tumors (30). Our GSEA analysis of the T-ALL cDEG revealed an upregulation of genes associated with early and late responses to estrogen, signatures that were defined as genes differentially modulated by 17ß-estradiol/E2 in cellular models (genesets refinements datasets: GSE15717,GSE36683, GSE5840,GSE9757, GSE2251, GSE2292, GSE26834, GSE8597). This correlated with the fact that E2 has a positive ES in CMAP. We can therefore formulate the reasonable hypothesis that T-ALL display a 17ß-estradiol/E2 constitutive gene expression profile that can be counteracted by α-estradiol to produce anti-leukemic effects. Whether α-estradiol acts by competing with 17ß-estradiol/E2 for ER receptors or by its own signaling remains to be determined.

NDGA/Masoprocol is exerting pleiotropic actions in many systems, particularly cancer cells. It is a phenolic ligand from the plant genus Larrea, used in traditional medicine in Mexico and SouthWest USA to treat many diseases as different as rheumatism, diabetes, pain, inflammation (31). NDGA has anti-oxidant and electrophilic properties and therefore has been reported to interfere with many cellular enzymes such as Lipoxygenases (LOX) but also with several tyrosine kinases (Insulin Receptor, HER2) or phosphatases such as PTEN or DUSPs.

We observed in our leukemic model that NDGA could interfere with the constitutive activation of mTORC1, an important driver of leukemic growth. Interestingly, NDGA was also shown to counteract mTORC1 activation in breast cancer cells leading to the death of cancer cells, both *in vitro* and in a xenograft model (32). Although not completely proven in that study, NDGA was proposed to act directly by disrupting mTORC1-Raptor interactions or indirectly, by stimulating the AMPK-TSC-2 pathway that counteracts mTORC1. We also observed that NDGA strongly decreased the cellular levels of the c-MYC oncogene which is overexpressed in many cancers including acute leukemia. Interfering with its expression could be an important feature of the anti-cancer effects of NDGA. Whether NDGA acts at transcriptional or post-translational levels remains to be more thoroughly studied.

We also identified prochlorperazine (PCZ) as a potentially active molecule on T-ALL. PCZ is analogous to chlorpromazine and therefore an antagonist of dopaminergic D2 receptors, explaining its antipsychotic and antiemetic effects and its current use during chemotherapy treatments (33). D2 receptors have been detected at mRNA and protein levels in a variety of cancer cells (acute myeloid leukemia, breast, ovarian, glioma, neuroblastoma), frequently correlating with tumor grade as well as a poor patient’s survival (cervical, esophageal, lung). Several pharmacological screening studies identified D2R antagonists as potential anti-cancer molecules, that interfered with FOXO localization or AKT activation. Another HTS identified PCZ as interfering with migration and metastasis of ovarian cancer cells in an *in vitro* 3D culture model (34). Moreover, a shRNA-based screen for glioma growth demonstrated the pro-tumoral role of D2R, further underlined by the fact that several antagonists interfered with *in vitro* growth of glioma cells (35). If the literature suggests that D2R antagonism could exert anti-cancer effects by inducing autophagy, apoptosis and proliferation arrest, the fact that the doses that are used in many studies are above the K_D_ of the receptor, suggests off-target effects and more studies to get definite proofs are required. Interestingly, PCZ was shown to inhibit dynamin-dependent endocytosis of membrane proteins such as EGFR, PD-L1 or HER2 which are the targets of therapeutic antibodies in several cancers. Preventing endocytosis of these proteins increased ADCC therapeutic effects (36). These effects seem independent on interference with dopamine, although D2R inhibition was also reported to increase anti-tumor immunity of cytotoxic T cells (37). In the EGFR model, PCZ was shown to interfere with AKT activation downstream of the receptor to decrease proliferative signaling. In our T-ALL model, PCZ was also observed to interfere with mTORC1 activation which is downstream of AKT and also to lower c-MYC levels.

α-E and NDGA and PCZ at a lower level, appeared to trigger a DNA damage response which could be part of their anti-leukemic activity.

In future experiments, it would be interesting to analyze both *in vitro* and *in vivo* if these three candidate anti-leukemic molecules could produce adjuvant effects when combined with existing therapeutic drugs for T-ALL, in particular dexamethasone and anthracyclines.

The use of a PTEN-deleted murine T-ALL model could be seen as a bias for a limited number of T-ALL cases as *PTEN* mutations are found only in 15-20% of the cases. However, the downstream Akt/mTor pathway whose activity is restrained by PTEN is found activated in almost 90% of T-ALL suggesting that PTEN is inactivated by non-genetic means (10, 11). This suggests that most T-ALL cases could be sensitive to the three drugs we selected through CMAP. It was not possible to test whether T-ALL cell lines could display different sensitivity to the drugs, depending on their PTEN status (+: HUT78, PEER, vs mutated: Jurkat, MOLT-16) as in our experience we never detected any correlation between the PTEN status and differences in mTORC1 activation or sensitivity to metabolic inhibitors such as a blocker of the Essential Amino Acid transporter that affects constitutive mTORC1 and induced leukemic cell death (16). Such studies should be conducted with precisely genetically defined primary T-ALL samples and not immortalized cell lines that not only are highly divergent, because T-ALL is molecularly heterogeneous but also because they have likely accumulated over the years in culture, additional mutations/adaptations to be able to grow *in vitro* outside their microenvironement. While PTEN abnormalities are clearly associated with a poor outcome for childhood T-ALL (38), this bad prognosis is mitigated by concomitant NOTCH1 mutations (39). T-ALL also frequently express a constitutive activation of the IL7-JAK-STAT5 pathway, through activating mutations in the genes for IL7 receptor (40), JAK3 (41) or STAT5 (42). It would thus be interesting to analyze on a panel of T-ALL primary samples or PDX, the sensitivity of the different mutational patterns to the three drugs we identified here.

Altogether our study reveals the existence of conserved genes potentially composing the core of T-ALL pathogenesis and that the bioinformatic mining of genetic signatures from public datasets and experimental models could be exploited to set up a strategy to search for new treatment avenues in T-ALL.

## Data Availability Statement

The raw data supporting the conclusions of this article will be made available by the authors, without undue reservation.

## Author Contributions

RB: methodology, bioinformatic analysis, writing original draft, review, editing the manuscript. MN: biological experiments, review of the manuscript. CB: biological experiments, review of the manuscript. FR: supervision of bioinformatic analysis, review of the manuscript. VI: analysis of revised data, review of the manuscript. PR: clinical input, review of the manuscript. J-FP: conceptualization, supervision of the project, design, methodology, writing original draft, review, editing the manuscript. All authors contributed to the article and approved the submitted version.

## Funding

This work was supported by institutional funds from Inserm and a grant from INCa (PLBio 2016-162).

## Conflict of Interest

The authors declare that the research was conducted in the absence of any commercial or financial relationships that could be construed as a potential conflict of interest.
