# Supplementary material for: New Drug Repositioning Candidates for T-ALL Identified Via Human/Murine Gene Signature Comparison
Source: Front Oncol. 2020 Nov 9;10:557643. doi: 10.3389/fonc.2020.557643 (PMC7680901; doi:10.3389/fonc.2020.557643)
Supplement: Supplementary file 1 [file DataSheet_1.docx]

Legends to supplementary figures and tables

Supplementary figure 1:

Analysis of the effects of Tanespimycin/17-AAG and Netilmicin after a 48h treatment of the MOLT-16 human T-ALL cell line. Cell viability was measured by flow cytometry after DAPI staining. The figure represents the merge of three independent experiments. Results are presented as mean ± SD.

Supplementary figure 2:

This figure shows the effects of α-E on HL60 cells in CMAP on upregulated cDEG genes that are enriched in the hallmark estrogen response early (A) and in the hallmark estrogen response (B) late gene sets.
For each gene with duplicated probes, the average rank is represented. Bar colors show that the gene was either downregulated (blue) or upregulated (red) under α-E exposure. The red horizontal line corresponds to the average rank of control probes matching the HSP90AA1 housekeeping gene.

Supplementary figure 3:

Effects of α-E, NDGA and PCZ on cell death induction in MOLT-16 cells.

A: Apoptosis was measured by flow cytometry following annexin V-FITC / DAPI staining after 24h and 48h of incubation with or without 20µM of the pan-caspase inhibitor Q-VD-OPh. B: dot plot representation of the flow cytometry data displayed on A.

Supplementary figure 4:

Quantification of the protein bands displayed on the Western blots of Figure 6b,c. Quantification was performed using the ImagJ software. The signal of a given total protein was normalized to the signal of Hsp60, used as a loading control from the same sample. Bands corresponding to phosphorylated proteins were normalized to the signal of the corresponding total protein which had been normalized to Hsp60.

Supplementary figure 5:

Cell cycle analysis. MOLT-16 cells were incubated with the selected drugs at non toxic concentrations for 48h. Cells were then fixed and permeabilized with ethanol and incubated with propidium iodide before flow cytometry analysis. This experiment was repeated twice and at least 10 000 events were recorded. The histograms show the quantification of each cycle phase.

supplementary table 1:

List of differentially expressed genes between PTEN-KO vs WT (GSE39591), human T-ALL vs normal T cells (GSE48558), PHF6-KO (active NOTCH) vs WT (GSE117165).

supplementary table 2:

List of genes that are up regulated or down regulated in T-ALL cDEG.

supplementary table 3:

GO and fGSEA analysis of T-ALL cDEG. Hallmarks and Reactome pathways.

supplementary table 4:

Complete output table of molecules provided by CMAP applied to the T-ALL cDEG.

supplementary table 5:

Details of the bibliometric scores applied to the CMAP data obtained on T-ALL cDEGs.

supplementary table 6:

List of cDEGs, (both up and down) between GSE117165 (active NOTCH, PHF6-KO vs WT) and GSE48558 (human T-ALL vs normal T cells).

supplementary table 7:

CMAP results obtained using as a query the NOTCH1 cDEG (NOTCH1 oncogenic mouse model GSE117165, compared to T-ALL, GSE48558.

supplementary table 8:

Effect of α-E on genes composing the estrogen early and late response datasets. Score list.
