# Supplementary figures and images for: New Drug Repositioning Candidates for T-ALL Identified Via Human/Murine Gene Signature Comparison
Source: Front Oncol. 2020 Nov 9;10:557643. doi: 10.3389/fonc.2020.557643 (PMC7680901; doi:10.3389/fonc.2020.557643)

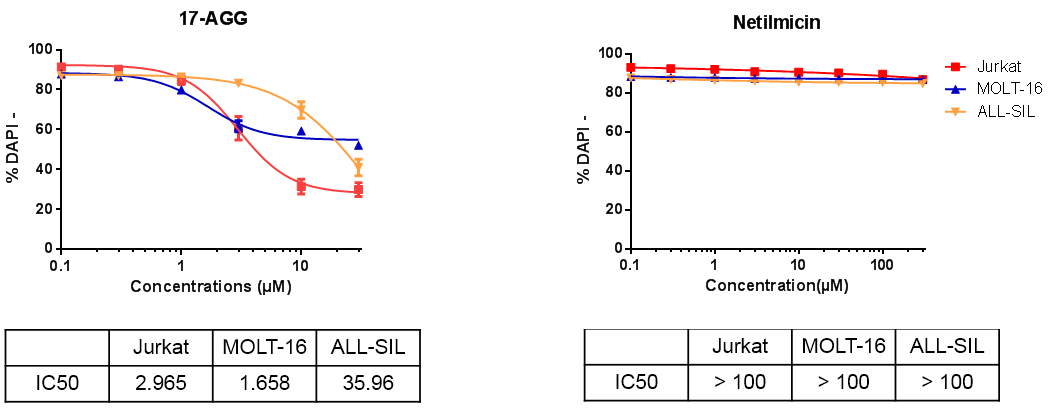

Supplement: Supplementary file 2 [file Image_1.png]

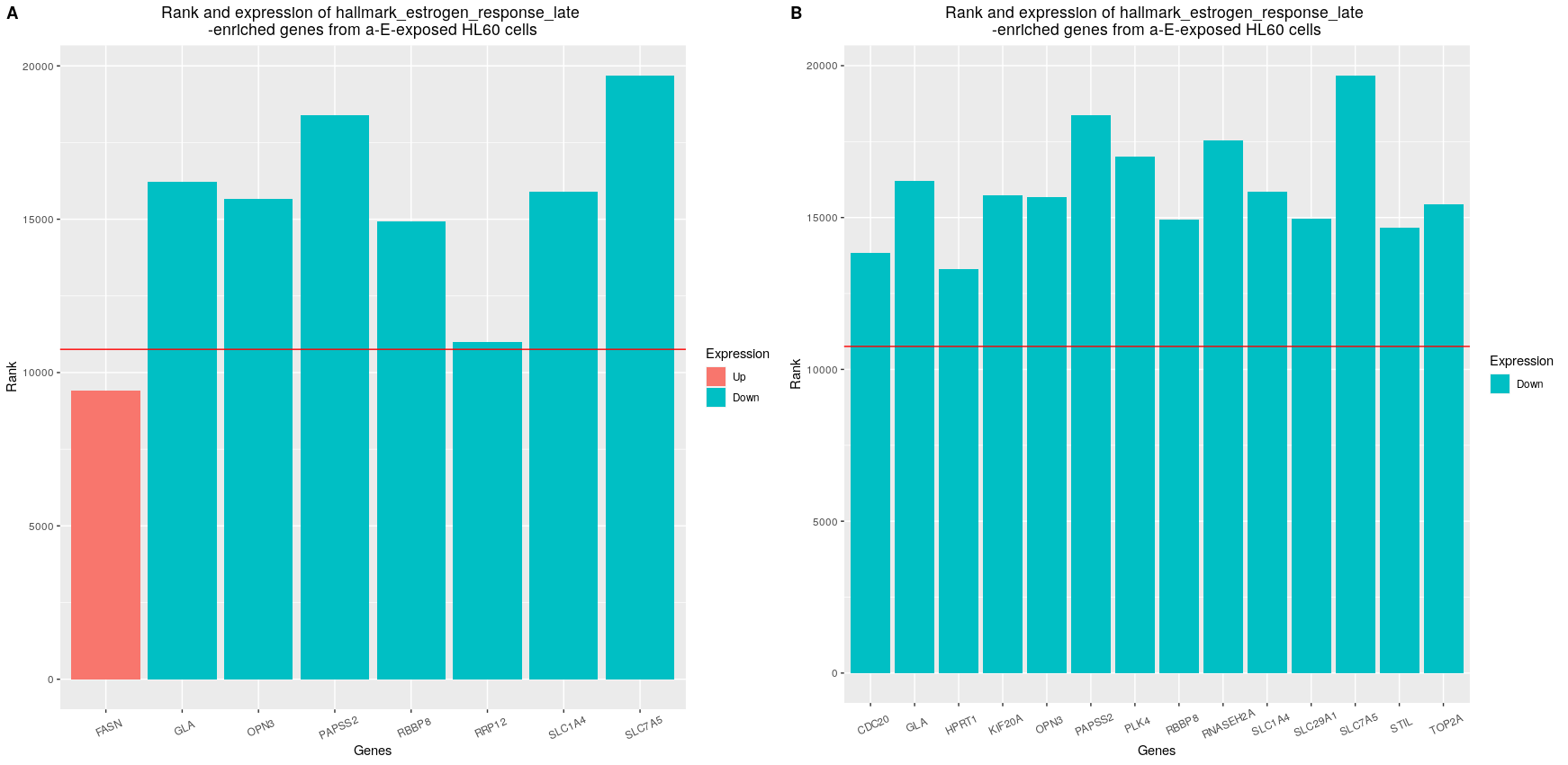

Supplement: Supplementary file 3 [file Image_2.png]

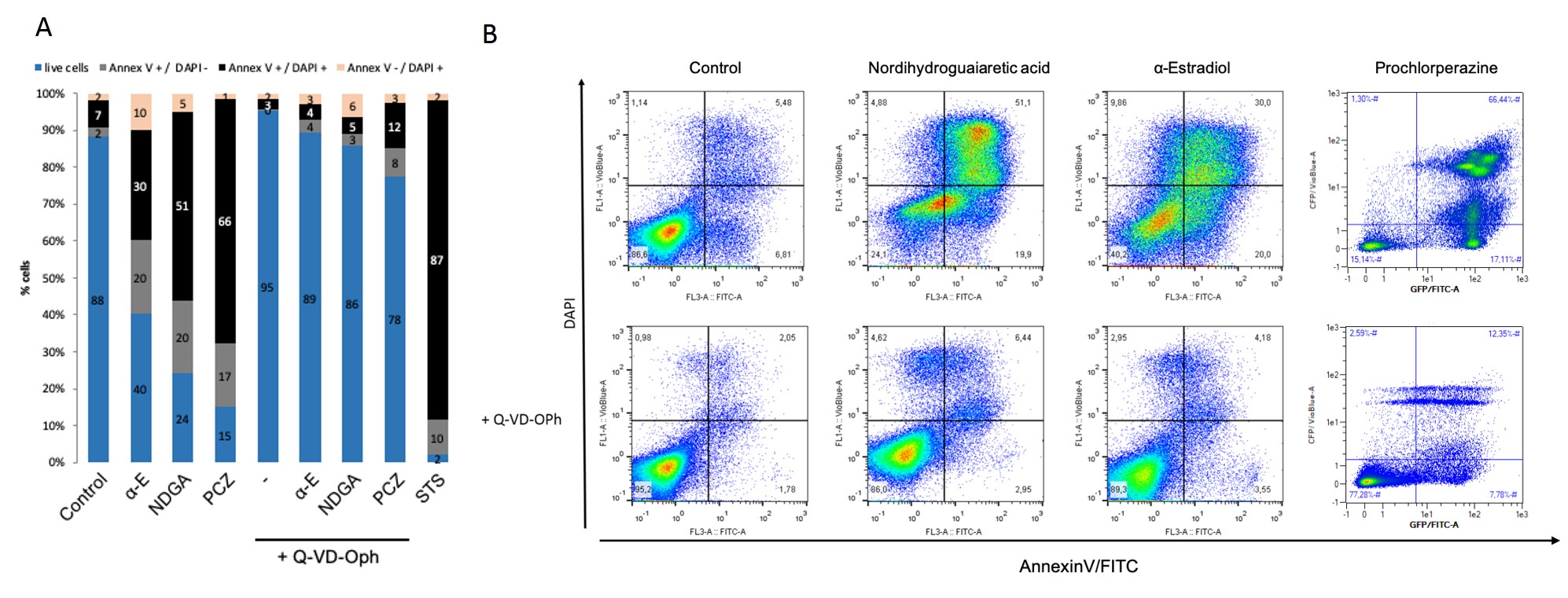

Supplement: Supplementary file 4 [file Image_3.jpeg]

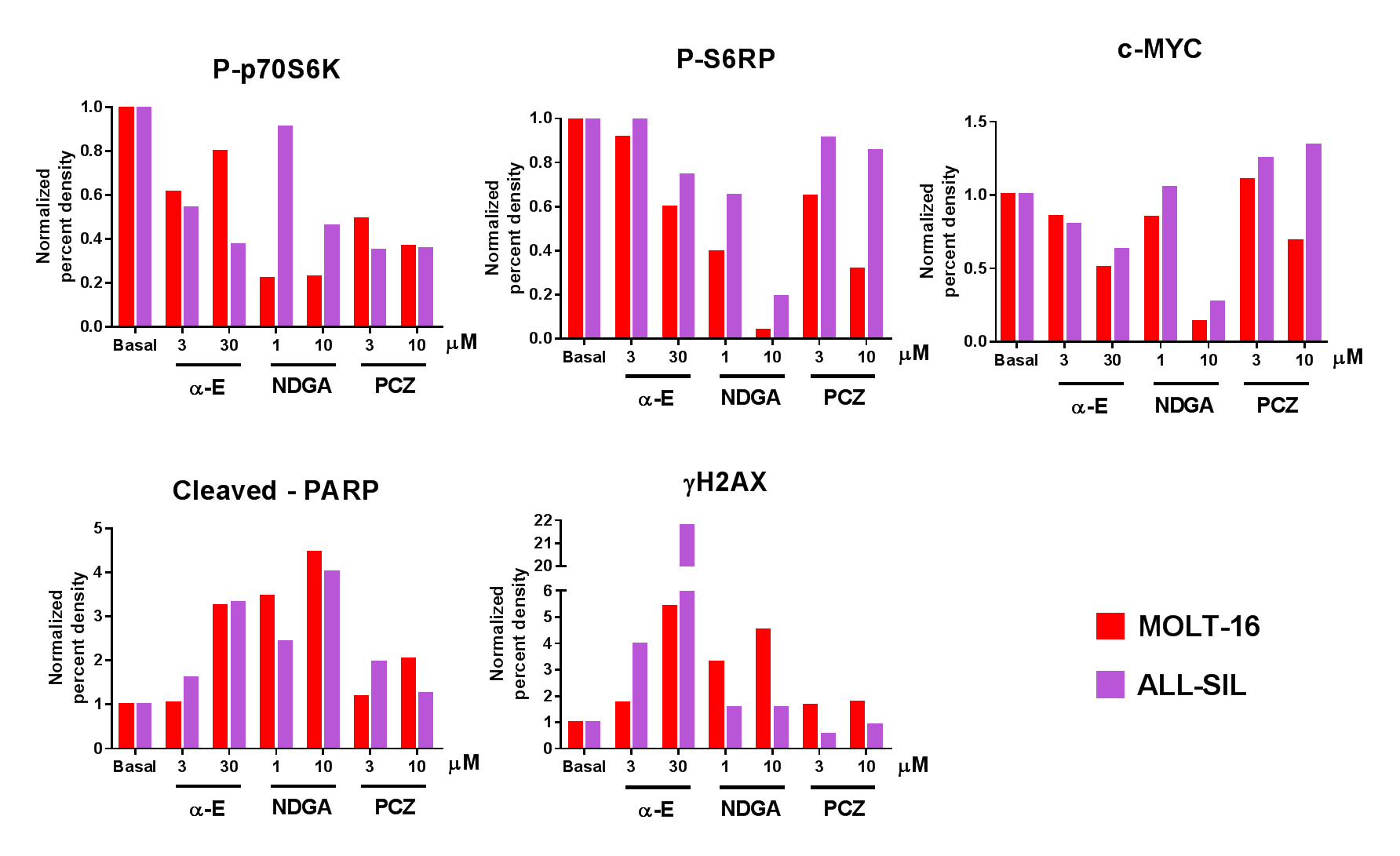

Supplement: Supplementary file 5 [file Image_4.tif]

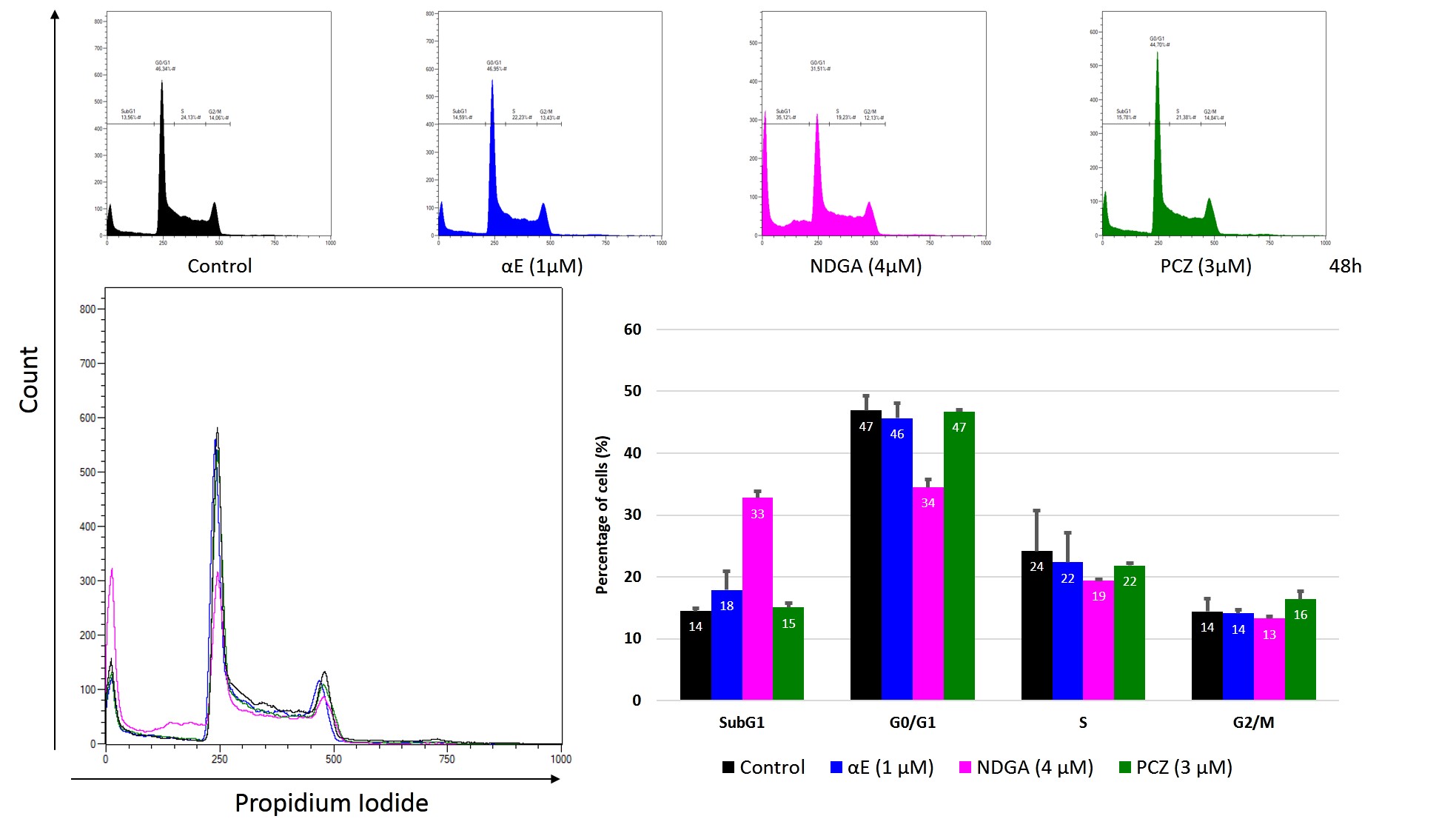

Supplement: Supplementary file 6 [file Image_5.jpeg]
